# A rare clinical image of a 16-year-old adolescent with juvenile spondyloarthritis

**DOI:** 10.11604/pamj.2025.50.95.46877

**Published:** 2025-04-08

**Authors:** Neha Ashok Brahmane, Sharath Hullumani

**Affiliations:** 1 Department of Paediatrics Physiotherapy, Ravi Nair Physiotherapy College, Datta Meghe Institute of Higher Education and Research, Sawangi (Meghe), Wardha, Maharashtra, India

**Keywords:** Bilateral hip arthritis, bilateral sacroiliitis, rehabilitation, juvenile idiopathic arthritis, juvenile spondyloarthritis

## Image in medicine

Juvenile spondyloarthritis is a chronic inflammatory condition that starts during childhood (before 16 years of age). Majorly characterizes arthritis, HLA-B27 positively, sacroiliitis, and inflammatory bowel disease. The prevalence estimates range from 1-4, which is 1% of the population. A 16-year-old boy visited a tertiary care hospital with complaints of severe pain in the hip joint while at rest as well as during movement. According to the primary caregiver, the child had a history of a fall from a bicycle on the right side of the body 5 years ago. The pain was progressive and later affected the activities of daily living. The magnetic resonance imaging (MRI) scan indicated bilateral hip arthritis with bilateral sacroiliitis. Subsequently, the patient underwent conservative treatment including intravenous antibiotics, antacids, antiemetics, analgesics, and various supportive treatments. For further rehabilitation, a structured physiotherapy program focused on pain management through early mobilization, reach-out exercises, and gait training. The case highlights the importance of combining medical treatment with structured physiotherapy rehabilitation to inhibit pain, optimize activities of daily living (ADL´s), enhance recovery, and improve patient´s well-being.

**Figure 1:**
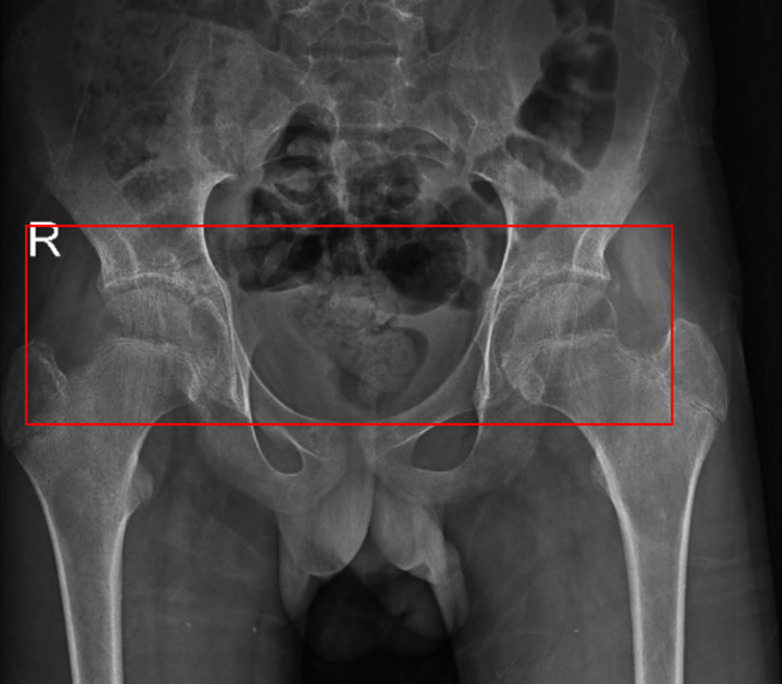
magnetic resonance imaging scan indicated bilateral hip arthritis with bilateral sacroiliitis, T2/PDFS hyperintensity was noted in the bilateral sacroiliac joint, erosion of bilateral acetabular surface, minimal bilateral hip joint effusion with marginal erosion along the articular surface, T2/PDFS hyperintensity was noted in the neck of the femur and greater trochanter of bilateral femur S/O edema

